# Progress in Bacteriology

**Published:** 1902-06-21

**Authors:** 


### PROGRESS IN BACTERIOLOGY.

(*Continued from page* 189.)

**Streptococci.**—Marmorek[7] states that the two most constant features of the many varieties of streptococci are, the clear dew-like growth on solid media, and the preservation of the rounded form in chain series. He concludes that all the streptococci pathogenic to man belong to the same species, and that there is no ground for the present tendency to differentiate them on morphological or toxicological characteristics. With the exception of the streptococcus of scarlatina, they all possess marked hæmolytic powers which are proportionate to their virulence. Streptococci will not grow in a filtrate of the broth medium in which they have for some time developed, and one variety of streptococcus will not grow in the filtrate in which a streptococcus taken from an entirely different source has grown, although other organisms can flourish in the same. A properly prepared antistreptococcic serum will protect against each and every sort of streptococcus. The differences which exist between various cultures of streptococci are differences of quantity and not of quality. The organisms associated with scarlatina and glanders however, seem to stand apart in the series. Marmorek's conclusions are based on observations made on 42 types of streptococci drawn from different sources. In a second paper in the same issue Marmorek announces that he has successfully prepared and isolated the streptococcus toxin, and that this is the same for all varieties. These observations are of especial interest in connection with anti-streptococcic serum therapy, for they show that, provided the infecting organism be a streptococcus, it is a matter of indifference from what source the antitoxin may have been prepared, the use of "polyvalent" antitoxins being unnecessary. The significance of the presence of streptococci in drinking-water is insisted upon by Houston.[8] These organisms are of low vitality, and quickly die; they are present in human fæces and in crude sewage, and in waters contaminated by sewage. Virgin soils and pure waters never contain streptococci, nor do polluted soils and waters unless the contamination be recent. For these reasons Houston regards the detection of streptococci as one of the most delicate tests of recent dangerous contamination of drinking-water. Streptococci may be detected in $\frac{1}{1000}$c.cm. of crude sewage, and should be absent from 10c.cs. of pure water.

**Micro-organisms in Milk.**—In a review of the subject of pathogenic organisms in milk Bergey[9] is quoted as finding micrococci in 90 per cent. of samples and streptococci in 50 per cent. In first-class dairies the percentages were much lower, but there can be no doubt that these organisms are present sufficiently frequently to cause many outbreaks of gastro-enteritis. Klein in 150 samples found B. tuberculosis in seven, B. pseudo-tuberculosis in eight, B. diphtheriæ in one, and a pathogenic torula in another. The chief points governing the quantity of germs in milk are—(1) the original amount of germicidal substance; (2) contamination on and after milking; (3) lapse of time since milking; (4) temperature during storage. If the milk be kept at a temperature of 50° F. or lower the multiplication of bacteria is delayed for twenty-four hours. In New York, Park found 300,000 bacteria per c.cm. of milk in cold weather, in cool weather 1,000,000, and in hot weather 5,000,000. In the best shops where careful storage is carried out the numbers of bacteria only varied between 10,000 and 30,000 per c.cm. As a standard in New York it has been decided to exclude all milk containing more than 500,000 bacteria per c.cm. from entering the city, and a milk containing 1,000,000 per c.cm. when delivered to the consumer is also to be condemned. The Pediatric Society of Philadelphia seek to fix the standard at 10,000 bacteria per c.cm.

**Acute Dysentery.**—Vedder and Duval[10] find that the various organisms described by Shiga, Flexner, Kruse, and Strong, associated with this disease are varieties of the same bacillus, and that the same germ is the infective agent in cases under their care in America, including many so-called sporadic "terminal dysenteries" and cases occurring in asylum epidemics. This organism the bacillus dysenteriæ of Shiga has the following morphological and cultural characteristics: it is a slender rod 1 to 3 $\mu$ in length, staining with all the aniline dyes, but decolorised by Gram; there is sometimes a capsule, and flagellæ are numerous. Motility has not been noted. Although the organism is fatal when injected intraperitoneally into guinea-pigs, it has not been found possible to induce the symptoms of disease in these animals, but Strong feeding cultures to a Filipino prisoner induced an attack of acute dysentery. The various strains of B. dysenteriæ show differences of colour and rapidity of growth, but all grow well on agar, forming colonies very similar to the B. coli or B. typhosus. Gelatin is not liquefied, and a dense sediment falls in broth cultures. In litmus milk, acid is formed, but there is no tendency to coagulation.

[7] An. de l'Inst. Past., March 25, 1902. [8] Brit. Med. Jour., Dec. 21, 1901. [9] Public Health, Feb. 1902. [10] Jour. of Exper. Med., Feb. 5, 1902.

